# Assessment of blood sampling time points to determine the relative bioavailability of ruminally protected methionine supplements using the plasma free amino acid dose-response technique

**DOI:** 10.3168/jdsc.2023-0508

**Published:** 2024-03-29

**Authors:** Nancy L. Whitehouse, Devan L. Chirgwin, Charles G. Schwab, Daniel Luchini, Nelson Lobos, André F. Brito

**Affiliations:** 1 Department of Agriculture, Nutrition, and Food Systems, University of New Hampshire, Durham, NH 03824; 2 Schwab Consulting LLC, Boscobel, WI 53805; 3 Adisseo USA Inc., Alpharetta, GA 30022; 4 Pioneer Hi-Bred International Inc., Johnston, IA 50131

## Abstract

The calculation of the relative bioavailability (RBV) of rumen-protected AA supplements using the plasma free AA dose-response technique currently relies on blood samples obtained 2, 4, 6, and 8 h after the 0500 h feeding during the last 3 d of each period in Latin square experiments with cows fed every 8 h (0500, 1300, and 2100 h). The objective of this study was to determine if this current blood sampling protocol captures the changes that may occur in plasma Met concentrations within a 24-h day to adequately determine the RBV of Met from Smartamine M (SM). Five multiparous lactating Holstein cows were used in a 5 × 5 Latin square design with 7-d periods. Treatments were (1) control (abomasal infusion of tap water), (2) 12 g/d of abomasally infused dl-Met, (3) 24 g/d of abomasally infused dl-Met, (4) 15 g/d of fed Met (20 g/d of SM), and (5) 30 g/d of fed Met (40 g/d of SM). Blood samples were collected via jugular catheters every 2 h after the 0500 h feeding starting on d 5 and ending on d 7 of each period. Plasma Met analysis was conducted using gas chromatography after chloroformate derivatization. Plasma Met concentration was averaged across days for 2–8 h after the 0500 h feeding, 2–8 h after the 1300 h feeding, 2–8 h after the 2100 h feeding, and 2–24 h after the 0500 h feeding. In addition, plasma Met concentration was regressed on 0, 12, and 24 g of infused dl-Met and 0, 15, and 30 g of fed Met. The calculated RBV of Met from SM averaged 83.8%, 83.6%, 87.4%, and 83.0% for the 2–8 h, 10–16 h, 18–24 h, and 2–24 h sampling periods, respectively. The similarity in the estimations of RBV for the 2–8 h and 2–24 h sampling periods indicates that our original blood sampling protocol seems reliable for determining the RBV of ruminally protected Met products.

## Highlights

• Plasma Met concentration reached steady-state conditions by day 4.
• Plasma Met concentrations within an 8-hour sampling period did not differ from each other.
• The bioavailabilities for Smartamine M from the different blood sampling times were not different from each other.

## Graphical Abstract

**Figure:**
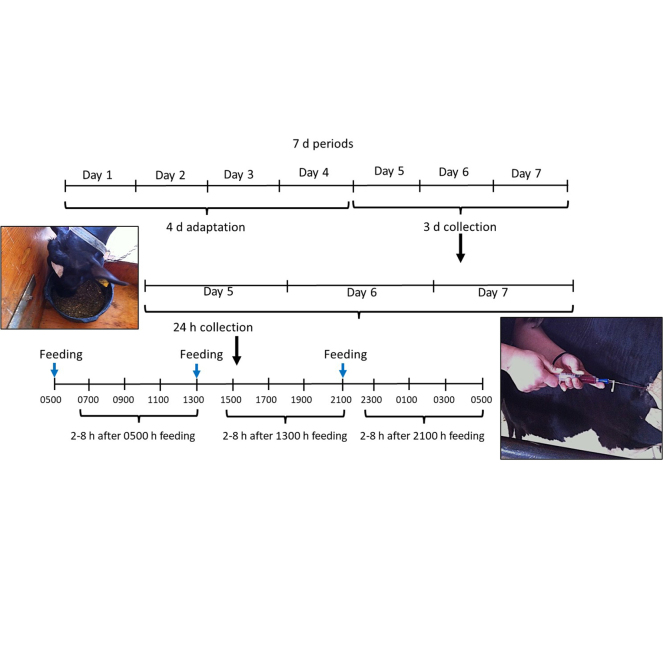
**Summary:** Estimating the relative bioavailability of rumen-protected (RP) amino acid supplements using the plasma free amino acid dose-response methodology currently uses blood collected at 2, 4, 6, and 8 hours after the 0500 hour feeding over 3 days, with cows being fed every 8 hours (0500, 1300, and 2100 hours). We conducted a study where blood samples were collected at 2, 4, 6, and 8 hours after each of the feedings to investigate whether this sampling protocol captures the diurnal changes in plasma methionine (Met) concentrations that may exist to adequately calculate the relative bioavailability of Met from RP-Met supplements. There was no diurnal variation in plasma Met concentration, and the relative bioavailability calculated for the RP-Met supplement tested did not differ significantly, thus indicating that the proposed sampling protocol can be used to determine the relative bioavailability of RP-Met supplements.

The plasma free AA dose-response technique has been used to determine the relative bioavailability (**RBV**) of rumen-protected (**RP**) AA such as Lys and Met (Chirgwin et al., 2015; Whitehouse et al., 2017). The technique has also been used to differentiate RP Lys products based on their RBV (Whitehouse et al., 2017). For instance, in a study reported in Whitehouse et al. (2017), the RBV of Lys of 3 RP Lys increased from 17.1 to 28.8% following modifications in the encapsulation matrix done by the manufacturer, with differences in RBV detected among the 3 RP-Lys supplements tested.

Our approach using the plasma free AA dose-response technique has been to feed 3 equal amounts of RP AA supplements every 8 h but only collecting blood at 2, 4, 6, and 8 h after the morning feeding during the last 3 d of a 7-d period in Latin square design studies (Whitehouse et al., 2017). Next, deproteinized blood samples from individual cows are pooled across the 4 daily time points, yielding 3 composited samples per cow per period for AA analyses and RBV calculations (Chirgwin et al., 2015; Whitehouse et al., 2017). However, it is possible that our proposed method may not capture the 24-h variation in plasma concentration of the AA of interest because of our feeding protocol or milking time, and that the calculated RBV may be biased when testing RP AA supplements that differ in rates of ruminal passage and intestinal release. For example, Smartamine M (**SM**; Adisseo France SAS) is known to have a rapid and complete release of Met in the abomasum (Kihal et al., 2021), whereas most of the other RP Met supplements currently available in the US market are advertised to have a slower and more continuous release of Met throughout the gastrointestinal tract. Another potential limitation is whether the current blood sampling protocol (i.e., 4 time points after the 0500 h feeding) provides unbiased estimations of RBV for different RP AA supplements or if additional blood collections are needed to better represent the absorption patterns and blood plasma appearance of AA from a variety of RP AA products.

We hypothesized that the RBV of Met supplied by SM would not be different across different sampling time periods with blood collection done throughout the day. Our objectives were to examine (1) the effect of sampling time (every 2 h for 24 h over 3 d) on plasma Met concentrations in dairy cows supplied with increasing amounts of Met via continuous abomasal infusion of dl-Met or feeding SM 3 times daily, and (2) if calculated RBV of Met from SM would be affected by the feeding period interval in which blood samples were obtained.

All procedures related to animal care were conducted with approval of the University of New Hampshire Institutional Animal Care and Use Committee (IACUC no. 140403). The experiment was conducted at the University of New Hampshire Fairchild Dairy Teaching and Research Center (Durham) from June 5 to July 17, 2014.

Five ruminally cannulated Holstein cows averaging (mean ± SD) 148 ± 59 DIM and 709 ± 88 kg of BW in the beginning of the study were fed a Met-deficient basal diet and randomly assigned to treatments in a 5 × 5 Latin square with 7-d experimental periods. The experimental treatments were (1) abomasal infusion of tap water (0 g/d of Met; control [**CON**]), (2) abomasal infusion of 12 g/d of dl-Met (**INF-12**), (3) abomasal infusion of 24 g/d of dl-Met (**INF-24**), (4) 15 g/d of fed Met (20 g/d of SM; **SM-15**), and (5) 30 g/d of fed Met (40 g/d of SM; **SM-30**).

Cows were housed in a naturally ventilated tiestall barn and fed individually 3 equal amounts of TMR at 0500, 1300, and 2100 h with minimal orts (2% to 4% of the as-fed intake). A Super Data Ranger mixer (American Calan Inc.) was used to make the TMR. Cows had access to water throughout the duration of the experiment and were milked twice daily at 0430 and 1530 h with milk weights recorded during each milking event. The basal diet contained (DM basis) 34% corn silage; 14.5% mixed, mostly grass silage; 4.6% steam-flaked corn; 8.7% citrus pulp; 11.9% ground corn; 3.6% soyhulls; 1.3% sugarcane molasses; 1.1% corn distillers grains plus solubles; 10.1% soybean meal; 3.2% canola meal; 0.2% urea; 2.2% ProvALL lysine (Perdue AgriBusiness); 1.4% Berga fat (80% palmitic acid; Berg + Schmidt America LLC); and 3.2% mineral and vitamin premix. The premix (as-fed basis) contained 42.5% calcium carbonate, 25.8% sodium sesquicarbonate, 12.3% sodium chloride, 9.5% magnesium oxide, 5.5% calcium phosphate, 0.5% mineral oil, 0.45% calcium sulfate, 0.28% vitamin E, 0.24% zinc sulfate, 0.18% zinc methionine complex, 0.17% manganese sulfate, 0.16% Rumensin 90 (monensin sodium; Elanco Animal Health), 0.09% Co, 0.07% biotin, 0.06% soybean oil, 0.06% ferrous sulfate, 0.06% Mn, 0.04% Cu, 0.017% Zn, 0.012% vitamin A, 0.007% vitamin D, 0.003% cobalt sulfate, and 0.001% calcium iodate. Samples of TMR and orts were collected daily and composited by equal weight in each period for determination of DM, which averaged 42.2%. The chemical composition of the basal diet averaged (DM basis): 16.9% CP, 31.4% NDF, and 20.6% ADF and provided 44 g/d of MP-Met and 171 g/d of MP-Lys according to the NRC (2001).

The infusion solutions of Met were prepared daily at 1300 h by mixing 4 L of hot tap water with dl-Met (Adisseo France SAS) followed by continuous infusion into the abomasum via the ruminal cannula using a peristaltic pump (Masterflex; Cole-Parmer) with the apparatus reported by Gressley et al. (2006). The pump was turned off and the infusion lines disconnected twice daily during the milking times for up to 45 min per milking. Pumping rate (175 mL/h) was adjusted and monitored daily to ensure uniform and complete administration of dl-Met. The amounts of SM fed daily (15 and 30 g) were divided into 3 equal aliquots and mixed with 1 kg of TMR, which was placed in rubber tubs and offered 30 min before feeding the remaining portion of TMR. If not totally consumed within 15 min, leftovers of the TMR-SM mix were administered through the ruminal cannula.

Cows were fitted with jugular indwelling Tygon catheters on d 4 of each experimental period between 0900 and 1100 h for blood collections. Starting on d 5 at 0700 h, blood samples were collected every 2 h for the next 72 h. With this sampling protocol, we intended to collect 36 blood samples per cow per period (n = 900 total). However, only 702 blood samples (i.e., 78% of the total planned) were collected and analyzed for plasma Met concentration due to catheter malfunctions (i.e., blockage by blood clotting, loss of patency) that occurred mostly on d 6 and 7 between 1700 and 0800 h when the veterinarian was not available to make replacements. Blood samples were collected using a 10-mL syringe and transferred into 10-mL Vacutainer tubes containing 15% EDTA (Monoject). Tubes were placed immediately in a Chameleon Cooler (Fisher Scientific) and centrifuged within 15 min at 1,200 × *g* for 20 min at 5°C. One milliliter aliquots were transferred to 1.8-mL cryovials and stored at –80°C for Met analysis done by GC after chloroformate derivatization using the EZ:Faast kit (Phenomenex) at the USDA ARS Dairy Forage Research Center (Madison, WI).

The UNIVARIATE procedure of SAS (version 9.4; SAS Institute Inc.) was used to identify outliers within cow and treatment. An observation greater than 2.0 SD from the mean was considered an outlier, thus resulting in 43 outliers that were detected and removed from the final dataset. Therefore, the final dataset consisted of 137, 126, 113, 144, and 139 observations for the CON, INF-12, INF-24, SM-15, and SM-30 treatments, respectively. A statistical distribution analysis of the data (NORMAL option in the UNIVARIATE procedure of SAS) showed a normal distribution of values across all time points. Plasma Met concentrations for individual cows were analyzed using the MIXED procedure of SAS with REPEATED measurements for day and time (i.e., hour) according to the following model:

Y_ijkl_ = μ + L_i_ + P_j_ + H_k_ + D_l_ + HD_kl_ + LH_ik_ + LD_il_ + LHD_ikl_ + E_ijkl_,

where Y_ijkl_ = dependent variable, μ = overall mean, L_i_ = fixed effect of the ith Met level, P_j_ = fixed effect of the jth period, H_k_ = fixed effect of the kth hour, D_l_ = fixed effect of the lth day, HD_kl_ = fixed effect of the interaction between the kth hour and the lth day, LH_ik_ = fixed effect of the interaction between the ith Met level and the kth hour, LD_il_ = fixed effect of the interaction between the ith Met level and the lth day, LHD_ikl_ = fixed effect of the interaction among the ith Met level, the kth hour, and the lth day, and E_ijkl_ = random residual error $∼N\left(0,\sigma_{e}^{2}\right).$ The random effect of cow was used as the error term in the model. Degrees of freedom were calculated using the Kenward-Roger option of the MIXED procedure of SAS. For REPEATED measurements, the following covariance structure matrices were tested: compound symmetry, unstructured, and autoregressive 1. The covariance matrix with the lowest Bayesian information criterion was autoregressive 1, which was retained in the final model. Significance was declared at *P* ≤ 0.05 and tendencies at 0.05 > *P* ≤ 0.10. Linear and quadratic effects were determined using the CONTRAST option of SAS. The effect of day, and the interactions hour × day, Met level × day, and Met level × hour × day showed *P*-values of 0.50, 0.65, 0.43, and 0.97, respectively, and were removed from the final model. The plasma concentrations of Met were then averaged across the 3 d of sampling and rerun with a reduced model using the MIXED procedure of SAS. The linear and quadratic *P*-values generated by the MIXED model were <0.001 and 0.42, respectively. The resulting plasma Met concentrations were used in the MIXED procedure of SAS with the PDIFF option to obtain the LSM according to the following model to determine if there was a time effect:

Y_i_ = μ + T_i_ + E_i_,

where Y_i_ = dependent variable, μ = overall mean, T_i_ = fixed effect of the ith sampling time, and E_i_ = random residual error $∼N\left(0,\sigma_{e}^{2}\right).$ The random effect of cow was used as the error term in the model. Degrees of freedom were calculated using the Kenward-Roger option of the MIXED procedure of SAS.

The effect of time was not significant (*P* = 0.65). Therefore, we generated 4 time interval measurements to express the plasma concentration of Met as follows: 2–8 h after the 0500 h feeding, 2–8 h after the 1300 h feeding, 2–8 h after the 2100 h feeding, and 2–24 h after the 0500 h feeding. These measurements of plasma concentration of Met were then subjected to the REG procedure of SAS to obtain the slopes, regression variables, and R^2^ for the abomasal infusion of dl-Met and fed SM. The slopes of plasma Met versus grams of infused dl-Met or grams of fed SM were used to calculate RBV according to the following equation: RBV, % = (slope of fed SM/slope of dl-Met infused) × 100. The linear regression variables for the infused dl-Met and fed SM, and the respective RBV, as calculated using the individual and combined sampling periods, are shown in Table 1.

**Table 1:** Linear regression variables for abomasal infusion of dl-Met and fed Smartamine M (SM), and relative bioavailability (RBV) and 95% CI for SM as determined in the individual and combined sampling periods

Item^1^	Infused dl-Met	Fed SM
2–8 h after 0500 h feeding		
Slope	1.37^2^	1.15^3^
SEM of slope	0.05	0.04
R^2^	0.98	0.99
RBV, %	—	83.8^4^
CI	—	77.3–90.2
2–8 h after 1300 h feeding		
Slope	1.38^2^	1.16^3^
SEM of slope	0.05	0.04
R^2^	0.98	0.98
RBV, %	—	83.6^4^
CI	—	77.1–90.1
2–8 h after 2100 h feeding		
Slope	1.37^2^	1.20^3^
SEM of slope	0.06	0.04
R^2^	0.98	0.99
RBV, %	—	87.4^4^
CI	—	79.0–95.8
Within a 24-h period		
Slope	1.37^2^	1.16^3^
SEM of slope	0.05	0.03
R^2^	0.98	0.99
RBV, %	—	84.8^4^
CI	—	80.6–89.0

1 Blood samples were collected every 2 h for 3 d and analyzed Met concentrations pooled across day before statistical analysis.

2 Slopes for the infused dl-Met treatments were not different from each other (*P* = 0.97).

3 Slopes for the SM treatments were not different from each other (*P* = 0.75).

4 RBV estimates, calculated as RBV = (slope of SM fed/slope of dl-Met infused) × 100, were not different from each other (*P* = 0.82).

Figure 1 shows the diurnal variation in plasma Met concentration. Specifically, the diurnal variation was minimal, either within an 8-h feeding cycle or considering a 24-h feeding cycle, with no observed differences in the temporal patterns of infused dl-Met and Met supplied via SM (Met level × hour interaction; *P* = 0.87). Therefore, blood samples collected between 2 and 8 h after 0500, 1300, and 2100 h feedings appear to have adequately captured the diurnal variation in plasma Met concentration in response to different feeding times and changes in metabolic activity within a 24-h feeding cycle. However, we did observe a larger SE in plasma Met concentration for the INF-24 treatment (SE = 5.3 µ*M*) compared with CON (SE = 1.3 µ*M*), INF-12 (SE = 2.9 µ*M*), SM-15 (SE = 2.0 µ*M*), and SM-30 (SE = 2.7 µ*M*).

**Figure 1:**
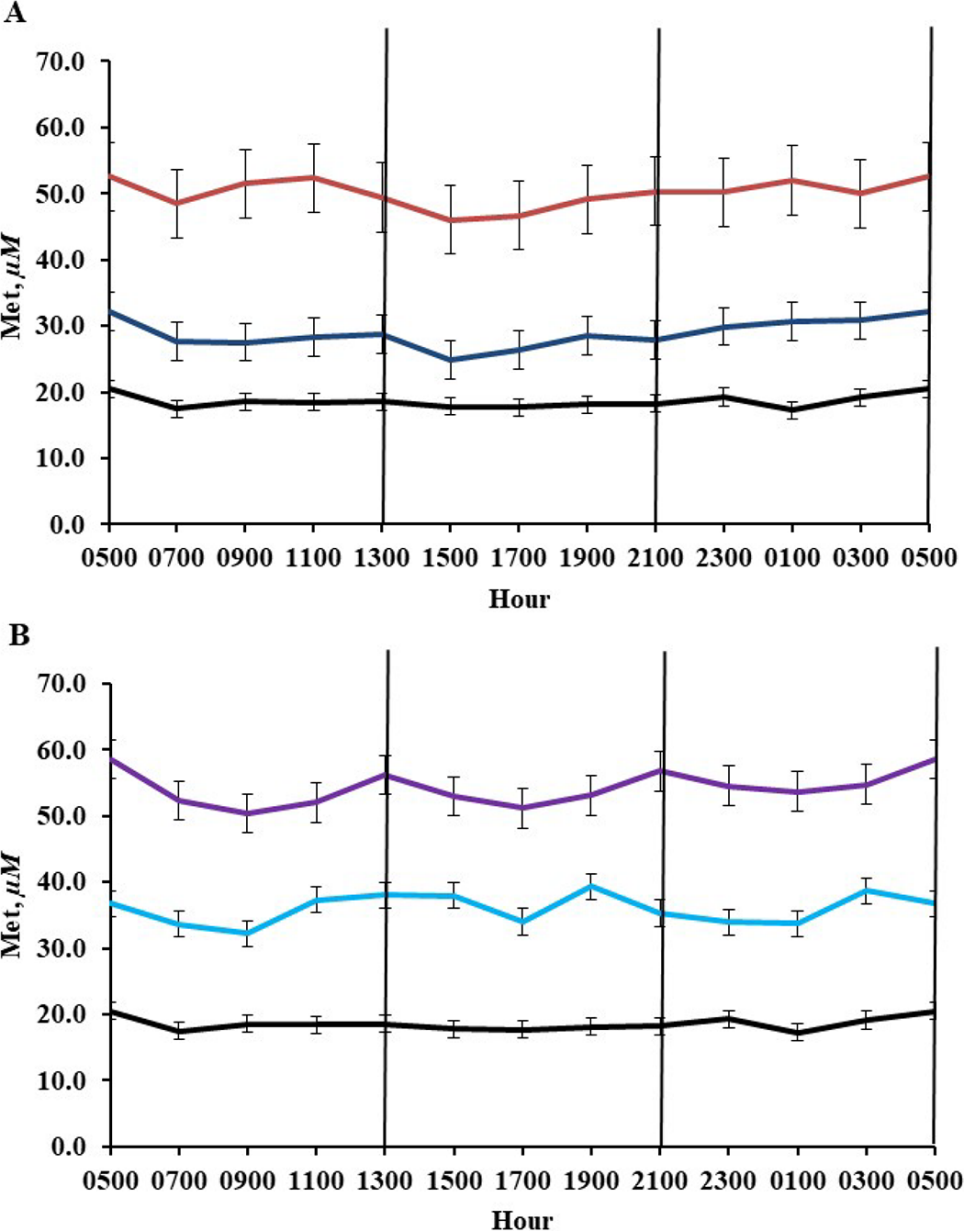
Effect of sampling day and sampling time on plasma Met concentration in lactating dairy cows fed a diet with increasing amounts of supplemental Met provided by abomasal infusion of dl-Met or by feeding Smartamine M (SM). Panel A shows the plasma Met concentration for the control treatment (black line), 12 g/d of infused dl-Met (dark blue line), and 24 g/d of infused dl-Met (red line). Panel B shows the plasma Met concentration for the control treatment (black line), 15 g/d of fed Met (20 g/d of SM; light blue line), and 30 g/d of fed Met (40 g/d of SM; purple line). The vertical lines indicate when cows were fed. Error bars indicate SE.

The calculated RBV of Met from SM averaged 83.8%, 83.6%, 87.4%, and 83.0% for the 2–8, 10–16, 18–24, and 2–24 h sampling periods, respectively. Also, we observed no effect of sampling day on plasma Met concentration for infusion of dl-Met or supplemental Met from SM (Met level × day interaction; *P* = 0.43), suggesting that 4 d of infused dl-Met or fed SM appear to be adequate to achieve steady-state conditions in plasma Met concentration. Our results agree with those from Rulquin and Kowalczyk (2003) who observed that 4 d of feeding SM was sufficient to obtain a steady state in plasma Met concentrations. Furthermore, Benefield et al. (2009) examined 7-d and 14-d period lengths and concluded that 7-d experimental periods were adequate in Latin square studies for measuring plasma Met concentrations when cows were fed different sources of RP Met. Even though the effect of day on plasma Met concentration was not significant, we recommend 3 d of blood collection to allow for appropriate analysis of outliers.

Overall, our proposed blood sampling protocol characterized by 4 collections at 2, 4, 6, and 8 h after the 0500 h feeding over 3 d, with cows being fed every 8 h (0500, 1300, and 2100 h), appears to be representative of any potential diurnal variation in plasma Met concentration due to similar RBV estimations of Met from SM across different sampling periods. Therefore, our comparative results of plasma Met concentrations obtained from cows receiving supplemental Met via continuous abomasal infusion or through fed SM indicate that our blood sampling, feeding, and milking schedules should also capture diurnal differences in plasma Met concentrations when testing other RP Met supplements that differ in gastrointestinal tract digestion characteristics. Additional benefits of our technique are that less blood sampling is needed, thus reducing labor and analytical costs while allowing for reliable estimation of RBV from SM and likely other RP Met supplements with different encapsulation technologies.
